# Childhood Trauma and Exposure to Violence Interventions: The Need for Effective and Feasible Evidence-Based Interventions

**DOI:** 10.3390/children10111760

**Published:** 2023-10-30

**Authors:** Petunia Tsheole, Lufuno Makhado, Angelina Maphula

**Affiliations:** 1 Department of Psychology, Faculty of Health Sciences, University of Venda, Thohoyandou 0950, South Africa; 2 Public Health, Faculty of Health Care Sciences, University of Venda, Thohoyandou 0950, South Africa; lufuno.makhado@univen.ac.za (L.M.); angelina.maphula@univen.ac.za (A.M.)

**Keywords:** child trauma, exposure to violence, interventions, PRISMA guidelines

## Abstract

Several crimes in South Africa cause physical, economic, and mental problems. Xenophobic attacks, mob justice, and other violent conduct directly traumatise children. Service delivery riots and physical and sexual abuse are examples. This evaluation evaluates childhood trauma and exposure to violence interventions. The review describes the therapeutic methods for traumatised children exposed to violence, the healthcare professionals administering them, and the strategies used to tailor the interventions. The researcher systematically searched PsycINFO, Google Scholar, PubMed, Science Direct, and EBSCOhost. Literature from 2011 to 31 July 2023 was searched, and 19 papers were chosen for further review after the systematic search. The authors conducted an eligibility evaluation according to PRISMA guidelines. A thorough review of article texts identified 19 papers that met eligibility standards. Only nineteen studies have validated trauma and violence therapies for children. An effective multi-phased intervention that is feasible and adaptable to varied socioeconomic backgrounds is needed. Further studies on the mental health benefits of brief trauma intervention treatment are needed.

## 1. Introduction 

According to the World Health Organization [1], children from 6 to 17 years are exposed to trauma and violence, which are pervasive public health issues in South Africa. In contrast to other African nations affected by war and natural disasters, the rates of trauma exposure among children are shockingly high [2]. Thus, 98% of South Africans are exposed to community violence, and sexual abuse amongst minors accounts for 54.2%, with increased rates of comorbidity noted [3]. In one study, nearly all adolescents with PTSD had comorbid disorders, predominantly dysthymic disorder and major depression [4]. Violence and trauma increase the risk of mental diseases, including post-traumatic stress disorder [5]. 

The prevalence of PTSD has been reported among poor urban adolescents. Childhood exposure to trauma and violence increases the probability of developing depression, anxiety, sleeping disorders, PTSD, and eating disorders [6]. Violence can be encountered through direct or indirect means. Direct exposure to violence pertains to the experience of being personally victimised [7]. Indirect exposure to violence, also known as vicarious exposure, relates to the observation, either directly or indirectly, of another individual being victimised [8]. Whether encountered firsthand or indirectly, violence manifests in various manifestations, encompassing physical, emotional, psychological, sexual, and verbal dimensions [9].

Irrespective of one’s victim, witness, or attacker role, empirical studies indicate that violence often leads to detrimental consequences. These consequences encompass a range of distressing experiences and maladaptive outcomes, both in the immediate aftermath and over an extended period [10].

According to research, 22.2% to 23.6% of minors have mental health issues [11]. Physical and emotional abuse, neglect, familial violence, and other forms of child abuse were strongly associated with sexual abuse [12]. The violence against minors, including sexual assault, domestic violence, and community violence, is abhorrent [13].

Child trauma and exposure to violence are strongly associated with drug abuse and potential dependence as a coping mechanism in adulthood [5,8,14]. Childhood trauma can profoundly affect adolescents’ mental health [11]. Furthermore, it has been found that childhood trauma and exposure to violence predict anxiety and depression later in life [14,15]. This is evidence that childhood trauma and exposure to violence is a particularly severe form of maltreatment, consistent with a larger body of research concerning “adverse childhood experiences” [16].

This issue may result in various disabling and expensive problems that pose difficulties throughout one’s lifetime. According to the World Health Organization [17], child trauma and exposure to violence are atypical experiences.

Due to the burden of care experienced at the community level, programmes encompass various initiatives that provide information, training, and skills development facilitated by non-governmental organisations (NGOs) and healthcare clinics. The utilisation of community health workers (CHWs) and lay counsellors as a means of addressing the scarcity of adequately educated healthcare professionals in the mental health field is a progressively favoured approach.

Evidence indicates that the accessibility of mental health treatments is limited in the Southern African region, as the number of mental health professionals per 100,000 individuals ranges from 0.05 to 1.52, in contrast to the estimated figure of 9.9 in Europe [17]. Children and adolescents in low–middle-income countries have a lower treatment prevalence of 159 compared to 664 adults per 100,000 population, according to the WHO [1].

The primary objective of this systematic review is to ascertain various interventions available for addressing African children’s mental health within community settings. Additionally, this research emphasises the self-reported barriers therapists encounter in implementing these interventions.

## 2. Methodology

The data were initially encoded to investigate aspects that are associated with the purpose of the study, including the type of intervention used, the degree of context that was addressed, and the reported mechanisms for successful implementation. Through induction, several supplementary codes were identified, encompassing various aspects such as psychological conditions linked to the intervention, the specific site where the intervention was implemented, and the individuals responsible for providing the intervention. 

### 2.1. Search Strategy

The study commenced by identifying 23,998 studies conducted between 1 January 2011 and 31 July 2023, sourced from PsycINFO, PubMed, Science Direct, Google Scholar, and EBSCOhost. To ensure the quality and relevance of the selected studies, the inclusion criteria were limited to peer-reviewed articles written in English. To refine the search effectively, the researchers employed a Boolean search strategy, a structured technique that permits users to combine or exclude terms to enhance search precision [18]. The Boolean search phrases were “Childhood and Trauma And Intervention” and “Childhood And Exposure To Violence And Intervention” (See Table 1). The initial database search yielded a total of 23,998 articles distributed across various sources, as follows: PubMed (1860), EBSCOhost (431), Science Direct (2987), Google Scholar (17,960), and PsycINFO (237). Subsequently, a systematic process was undertaken to filter and eliminate redundant and irrelevant articles. Initially, 16,540 duplicate entries were removed. Additionally, 4652 articles were eliminated through automated means, leaving a core set of 2806 articles for further scrutiny.

**Figure 1:**
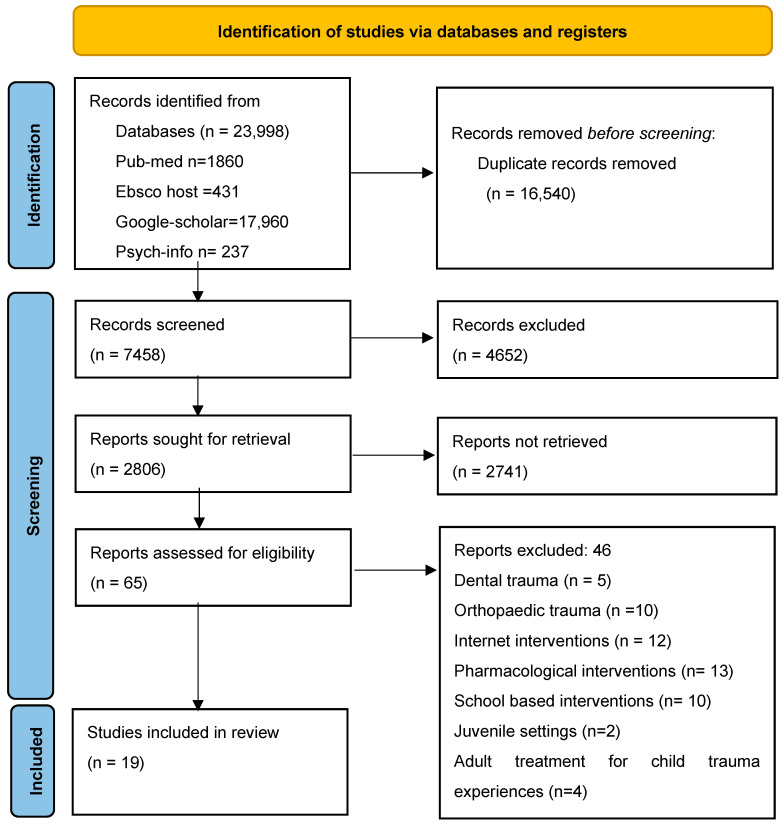
Prisma flow chart [19].

The subsequent phase involved a meticulous review of these 2806 articles. Three authors, PT, LM, and AM, independently assessed the articles based on their titles. Following this initial screening, 65 articles were selected for further evaluation, where the authors scrutinised the abstracts. Finally, a comprehensive full-text review was conducted for these 65 selected articles to determine their suitability for inclusion in the study. Upon completing the full-text review, 46 articles were excluded from the study due to a lack of relevance or non-compliance with the specified inclusion criteria. The rationales for exclusion encompassed articles that concentrated on topics such as dental trauma, orthopaedic trauma, internet-based interventions, pharmacological interventions, school interventions, juvenile settings, adult treatments for childhood trauma, geographic factors, mental illness, study design, or result criteria. Ultimately, the rigorous selection process included 19 articles (as presented in Figure 1) that met the established criteria and were deemed pertinent to the study’s objectives.

### 2.2. Inclusion Criteria

The researcher focused on the specific population of children exposed to trauma and violence who have been seen at healthcare facilities or trauma facilities for trauma intervention. Furthermore, the focus intervention of interest was childhood trauma intervention used to treat children exposed to trauma and violence. The study setting included any trauma or healthcare facilities from low- or high-income countries. The language of the articles reviewed was English. The review focused on papers that were addressing children aged 6–17 years of age. The review also focused on papers published from 2011 to 31 July 2023.

### 2.3. Exclusion Criteria

The studies that were excluded are those that addressed sexual abuse in isolation or specifically sexual abuse as one type of trauma. Most of the studies were excluded if the focus was on clinical diagnoses, such as depression or anxiety, with no history of trauma. Most of the studies were excluded if they only addressed children with behavioural problems with no subsequent trauma, juvenile medical settings, or the social services placements of children with trauma. Studies not conducted in a healthcare setting or by healthcare professionals were excluded. Studies focused on intellectual disability, substance abuse, or neurological problems were excluded. Irrelevant interventions, such as pharmacological interventions, were excluded. Interventions that addressed trauma-related medical examinations (i.e., brain injuries) were also excluded.

### 2.4. Data Extraction and Quality Assessment

For inter-researcher dependability, two authors (LM, AM) assessed the four papers to confirm data extraction uniformity. The remaining eight articles were split and removed. Author, date, geographic context, study population characteristics, sample size, intervention objective and design, mental disorder targeted, outcomes, results, and implementation facilitators and barriers were important. The essential data fundamentals that were abstracted from different studies were details about trauma-focused treatments aimed at children from 6 to 17 years and treatment outcomes. Details about treating possible mental health challenges and behavioural challenges due to trauma and exposure to violence amongst children were also abstracted. Finally, whether or not the main results of the studies indicated positive outcomes were recorded. Successful and ineffective interventions were included. Analysis of the extracted data was deductive and inductive. To investigate the study objective, trauma intervention, context addressed, and implementation mechanisms were coded. This study team (PT, LM, and AM) compared codes and cross-checked publications with uncertainty.

A critical appraisal checklist was used to assist the researcher in systematically assessing the reliability, relevance, and results to be issued. A critical appraisal checklist was used to assist the researcher in systematically assessing the trustworthiness, relevance, and results to be published [20]. The researchers utilised the CASP critical appraisal tool that consisted of ten questions. The questions were divided into three sections scored as “yes, can’t tell and no”. For each article appraised, the score was on a scale of 0–10 points by counting how many “yes”, “can’t tell”, or “no” responses there were. The articles that scored more than five “yes” responses were evaluated (See Table 2).

Of the nineteen studies selected, there were (n = 19) studies in the field under investigation that provided information regarding the outcomes of their interventions. Out of the total of 19 research studies examined, 3 of the studies indicated that TF-CBT was shown to be more feasible to train counsellors on. Three (3) studies on the different interventions used indicated positive results when used amongst children with TF-CBT. Four studies yielded statistically significant impact sizes, but the other two, randomised controlled trials (RCTs), showed non-significant effect sizes. The two systematic reviews indicated the need for a multimodal feasible intervention in one of the randomised controlled trials (RCTs) under consideration. The other indicated the positive impact of TF-CBT on treating children affected by trauma.

### 2.5. Characteristics of Included Studies and CASP Appraisal

The critical appraisal method was followed, explained as a procedure to carefully and systematically investigate research to critique its trustworthiness, value, and significance. The critical appraisal process involves selecting literature carefully and systematically as a tool that will be utilised for data analysis to assist the researcher in summarising the evidence found to judge its trustworthiness [40]. In applying the critical appraisal method, the researcher considered psychosocial interventions to manage trauma and exposure to violence among children (Table 3).

## 3. Methods

Based on the final search results, 19 papers were chosen as suitable for inclusion in the review. The majority of these papers were characterised by a quantitative [11] research approach with two qualitative studies [24,26], three systematic reviews [21,23,32], and two case studies [28]. According to Clarke [41], when there is significant variation in methodology, a systematic review can leave the findings of the studies to derive an average estimate. Consequently, the present study employed narrative synthesis to synthesise the data, employing thematic analysis as the analytical approach [42]. Textual analysis has been conceptualised as a technique for detecting, analysing, and reporting patterns or themes and searching for meaning within literature or data [43,44]. This study employed a six-step process for synthesising data through thematic analysis to identify recurring themes [45]. These steps included becoming acquainted with the data, creating initial (sub) codes, identifying (sub) themes, reviewing (sub) themes, and organising ideas or issues through charting [45].

### 3.1. Results

The thematic analysis yielded four themes (see Table 4), including “Effectiveness of childhood trauma intervention” [21,22,23,24,25,26,27,28,29], “Impact of trauma intervention” [22,26,28,29], “Barriers and facilitators of trauma” [24,25,29], “Coping strategies of children exposed to trauma” [21,22,24], and “Psychological burden of trauma” [22,26,27,29]. Table 5 provides key interventions reported to bear positive outcomes regarding childhood trauma management.

### 3.2. The Effectiveness and Impact of Trauma Intervention

PFA may be used for post-disaster therapies despite limited evidence. The iatrogenic effects of Critical Incident Stress Debriefing in adults render PFA unproven. PFA research is very early. Thus, while unlikely, negative impacts on children’s psychological functioning may still be found in Gilbert et al. [21]. The pilot intervention reduced PTSD and depression symptoms, although Woollett et al. [22] noted other benefits. Behavioural problems decreased. They understood, expressed, and controlled emotions through art and play. Finally, children and adolescents benefit from high-quality, systematic mindfulness therapy. These findings, paired with well-studied adult therapy, may reduce childhood trauma-related health problems. Ortiz et al. [23]. The lay counsellors stressed the necessity of Trauma-Focused Cognitive Behavioural Therapy (TF-CBT) in their community and cultural, belief, and socioeconomic sensitivity when working with participants. The authors also emphasised the importance of collaborating with various entities to effectively tackle socioeconomic obstacles [24]. The present study was unable to incorporate a comparison or waiting control group. The absence of a control group introduces ambiguity regarding the attribution of the outcomes to the specific components of TF-CBT or to non-specific factors such as the passage of time, the level of participation from the therapist, or the expectations associated with the treatment. This was further reiterated by [32], who indicated that three common problems in their findings were small sample sizes, the lack of a control group, and the lack of long-term follow-up. The examination of therapist–client relationships was not conducted in this study. These factors may have exerted an influence on results in conjunction with the impact of the treatment model. Therapists who conducted patient evaluations may have exhibited bias. The limited size of the sample hinders the ability to make generalisations [26].

Tabone et al. [27] examined CANS-ARC mapping in community-based clinical settings. The ARC framework and CANS assessed various traumatic symptoms in children and linked them to treatment planning and adjustment. After controlling for gender, race, age, guardianship, and service sites, the study indicated that children’s trauma-related symptoms improved considerably in all outcome categories. In their investigations, Fazel et al. [28] found that narrative exposure therapy (NET) is a child-specific therapy with expanding data. Lay or professional therapists tried it on refugees and asylum seekers in low, moderate, and high-income contexts. NET (or the child “KIDNET” version) therapy trials for PTSD in refugee children reveal that it is effective, scalable, and culturally adjustable. Dorsey et al. [29] found that TF-CBT can help children with PTS who lost a parent. However, results may vary. TF-CBT outperformed conventional care after therapy in urban Kenya, rural Kenya, and urban Tanzania, but not rural Tanzania. The 12-month follow-up showed condition discrepancies only in Kenya’s urban and rural districts. Secondary outcomes showed that TF-CBT had the greatest impact for rural Kenyan children after treatment and at 12 months for urban and rural Kenyan children.

### 3.3. Trauma Intervention Implementation

Kenyans and Tanzanians may have faced different hardships and adversity. Kenyan children and guardians had lower health, food deprivation, and higher stress. National statistics may have helped Kenyan children with typical care maintain symptoms during implementation [29]. Psychologists performed weekly online surveys documenting TF-CBT sessions with children and caregivers during the trauma intervention [26]. Psychologists recorded the following for each session: TF-CBT component(s) addressed, techniques/activities employed, skills taught, session length, barriers/challenges encountered, and next-session plans. Psychologists received self-reported feedback on treatment regimen compliance. To verify treatment model conformity, psychologists presented their TF-CBT cases during group consultation calls and received comments. This trauma profile matches childhood adversity in other low- and middle-income nations and is expected to be numerous [29]. Note the strong caregiver participation in treatment. TF-CBT relies on caregiver participation, which might be difficult in school-based child mental health treatment. Woollett et al. [22] used Art and play therapy with TF-CBT to produce a structured and client-led session. “Psychoeducation and parenting skills; Relaxation skills; Affective regulation abilities; Cognitive coping skills; Trauma narrative and cognitive processing of traumatic event(s) (PRACTICE); “In vivo mastery of trauma reminders; Conjoint child-parent sessions; and Enhancing safety and future developmental trajectory” constitute the TF-CBT acronym PRACTICE.

### 3.4. Barriers and Facilitators of Trauma

In a study conducted by Woods-jager et al. [24], Counsellors also discussed the difficulties in getting participants to articulate their feelings, noting that feelings are often unrecognised in their culture (for example, “Sometimes even sadness does not have a label in their language”). Counsellors pointed out that discussing death or speaking badly of the departed is usually not culturally acceptable. Steward et al. [26] indicated that the evaluators were the same therapists treating the patients, which could have created bias. Finally, the findings need to be more generalisable to other populations due to the small sample size. Witnessing participants’ unmet social and economic needs was noted as something that weighed on some counsellors and made TF-CBT implementation challenging at times [24]. According to Meetken et al. [25], it is an organised eight-phase technique to deal with the traumatic memory’s past, present, and future elements. During the sessions, a child is asked to choose a memory from a past hospitalisation that is currently the most traumatic to them. The painful thoughts are desensitised through regulated rhythmic eye motions, and pleasant and optimistic thoughts are programmed.

### 3.5. The Psychological Burden of Trauma in Children

According to Woollett et al. [22], violence harms children, affecting their mental, physical, and social well-being. Meta-analyses show that youth violence increases internalising and externalising difficulties. Steward et al. [26] also highlight El Salvador’s early challenges. Most children (90.7%) had experienced multiple stressful events. Most children experienced index traumas of domestic violence, physical abuse, and sexual abuse [26]. Trauma causes post-traumatic stress and other childhood mental and behavioural issues. Extensive literature relates childhood trauma to adult psychopathology and disability [44]. Childhood traumatic events cause mental distress in many persons who need mental health services, and the severity, frequency, and breadth of unpleasant experiences affect mental health. Childhood trauma is strongly linked to adult psychosis [11]. Ethnic minorities and the poor also endure more trauma. Most children (90.7%) had experienced multiple stressful events. Most children in the study encountered primary traumas such as domestic violence, physical abuse, and sexual abuse. This trauma pattern aligns with childhood adversity experiences observed in other low- and middle-income nations [29]. It is also consistent with expectations, given the prevalent exposure to armed conflicts, gang-related violence, and high homicide rates in many Central American countries [26,31]. It is important to note that employing standardised assessment data has its limitations. This is because attempting to condense the diverse challenges traumatised children face into a single diagnosis, such as post-traumatic stress disorder, may lead to misclassification or result in the assignment of multiple unrelated disorders [27].

### 3.6. Coping Strategies of Children Exposed to Trauma and Violence

Trauma exposure in early childhood may affect youth coping behaviours. Trauma may affect emotional and behavioural consequences by affecting coping skills. Self-portraits were used to examine coping resources and how children saw themselves and others. A comprehensive “Safe Place” fostered active conversation and planning [27]. Exposure to violence can result in sorrow, anxiety, thoughts of suicide, difficulties in peer relationships, and academic challenges [32]. Coping techniques are frequently integrated into social–emotional learning curricula, including school-based initiatives, and some have resulted in notable improvements in children’s well-being and academic performance from their early years through high school [33]. Vaughn-Coaxum et al. [33] underscore the significance of intervening during the school years when coping skills may be especially amenable to change. Wollet et al. [22] identify a practice guide that emphasises interventions to help children cope: “Psychoeducation and Parenting skills; Relaxation skills; Affective regulation skills; Cognitive coping skills; Trauma narrative and cognitive processing of the traumatic event(s); Conjoint child-parent sessions; In-person mastery of trauma reminders; and Improving safety and future development.”. PFA’s modularity is a benefit. Each module targets a distinct therapeutic aim (e.g., self-efficacy, coping skills, safety, connectivity) [21]. The first four groups taught children and guardians behavioural and cognitive coping techniques for parental loss and bereavement [24].

Table 5 illustrates that recent research has consistently demonstrated favourable results for children exposed to trauma through a range of therapeutic approaches, such as TF-CBT, child-centred play therapy, art-based therapy, narrative therapy, and psychoeducation [24,28,35,36,37,38,39].

## 4. Discussions

This review’s results indicated the limited research interventions conducted amongst children exposed to trauma and violence. The results obtained in this research offer validation for the suitability and acceptability of TF-CBT as an effective treatment method for enhancing the mental well-being of children. This aligns with Woollett et al. [22], who emphasised the significance of comprehending the strategies employed by non-specialist counsellors to ensure that the treatment remains culturally and socioeconomically relevant to the participants. This insight can inform the implementation of future Evidence-Based Treatments (EBTs) in an African context. Furthermore, these findings are corroborated by Copeland et al. [46], who reported that there was limited utilisation of mental health services by children and adolescents in programs not specifically designed for them. Moreover, the research underscores the importance of assessing interventions’ validity and cultural appropriateness in such contexts. Firstly, while the frequency of traumatic events is higher in post-conflict nations, the disparity of traumatic event categories differs significantly by location [47]. These findings reveal the effect of the socio-political and socio-economical context on the distribution of traumatic experiences [48]. Finally, we looked at new research suggesting how traumatic event exposure is becoming increasingly crucial in developing child mental health disorders. Most articles use more adopted interventions and lack cultural adaptability; however, according to Katsonga-Phiri [32], there is a significant increase in interventions adapted with input from community experts and local people regarding language and cultural relatability. The review further identified promising 21st-century therapies, particularly those utilising cognitive behavioural methods and cultural adaptations [32]. As indicated, both the methodology and results of adaptations should be documented and published to further this rapidly expanding study field. Other studies indicated positive results when using the interventions cross-culturally, although the techniques might not be effective due to cultural differences and contexts. Interventions to reduce the influence of traumatic event exposure and PTSD on the emergence of child chronic mental disorders are needed to address the high and growing burden of chronic mental health disorders among children.

The review results indicated the limited research interventions conducted for children exposed to trauma and violence in an African country as more focused on youth and indicated to be between 14 and 25 years [18,32,45]. Moreover, the validity and cultural reliability of the interventions are highlighted minimally. However, Coetzer et al. [49] highlighted the need for a multimodal intervention to address the challenges children might be experiencing and also, in their findings, they indicated the need for follow-ups, which were attributed to improved symptoms [50].

According to meta-analytic studies, it has been found that children in sub-Saharan Africa have greater prevalence rates of sexual, physical, and emotional abuse compared to other regions [51]. Specifically, around 83% of male and female children record instances of emotional abuse, while 64% report incidents of physical abuse [52]. Furthermore, it has been reported that 19% of boys in this region have experienced childhood sexual abuse [53]. There is evidence to show that male children in sub-Saharan African settings may face a greater susceptibility to physical abuse [54]. However, the factors contributing to this phenomenon are not well comprehended, and it should be noted that not all research indicates disparities in abuse rates based on gender [55].

The investigation of this subject holds significant importance, especially in sub-Saharan Africa, where peri-urban people face notable challenges such as extreme material poverty, inadequate infrastructure, and environmental risks [56]. Material deprivation can impact mental well-being via mechanisms such as experiencing financial difficulties, living in deteriorated neighbourhood environments, or facing limited opportunities [57].

Individuals who endured childhood physical abuse had lower life satisfaction, especially when combined with interpersonal violence [58]. Despite global efforts to eradicate violent discipline, low-income countries still witness the physical and psychological abuse of children under five [59]. This underscores the need for research on childhood exposure to violence interventions involving caregivers [60]. Interventions should address how children and adolescents interpret parental information and whether aggressive behaviour is reactive or intentional [61].

Studies show less physical violence by adolescents against parents in families with strong cohesion but more violence in families with high conflict [62]. Healthcare practitioners must enhance their capacity to recognise and address mental health issues to secure future generations’ well-being, using validated evidence for timely interventions [63]. Further research is essential to comprehensively understand caregiver and child victimisation [64]. This can enable social workers to collaborate with families and communities for effective interventions. Community violence can foster pessimism, disrupt social bonds, alter societal norms, and exacerbate violence frequency and trauma symptoms [65].

## 5. Evidence-Based Recommendations

A multi-phased and multi-modal intervention that encourages participants’ emotional needs to be expressed in a fluid and flexible manner is needed, advocating for additional studies into the effects of shorter treatments that impact positive mental health outcomes.

The intervention must guide the therapist and participant while permitting self-expression and the creative mastery of complex themes. The results of this review indicate the need for an intervention advocating for greater study on the influence of shorter impactful treatments on mental health and the involvement of stakeholders who can also be psycho-educated about trauma and its effects to ensure sustainability and empowerment. A phase-based approach that integrates techniques from different interventions to ensure that it fosters flexibility and the fluid expression of participant’s emotional needs while ensuring cultural sensitivity will guide the therapist and participant while allowing self-expression and the creative mastery of complicated issues.

Advocating for further investigation on the efficacy of abbreviated therapies and their effects on mental well-being encourages self-expression and creative problem-solving while maintaining cultural sensitivity and flexibility in meeting participants’ emotional needs. While many therapies have demonstrated adaptability, there remains an issue regarding the methodologies employed and their cultural acceptability across different societies. Moreover, this analysis highlights the necessity for further investigation to inform the execution and clinical results of interventions, specifically focusing on developing and customising interventions for children who have experienced trauma and violence.

## 6. Conclusions

This process highlighted five topics and interventions targeting a child’s underlying mental health due to trauma and exposure to violence. Three primary themes emerged: the effectiveness and implementation of trauma interventions, trauma intervention implementation, the barriers and facilitators of trauma, the psychological burden of trauma in children, and the coping strategies of children exposed to trauma. TF-CBT is the most used and most effective intervention that yields improved symptoms in children and youth, although the youth in most studies were around 19–25 years [14,66].

Informal settlements can worsen mental health difficulties because of the swift transformations in social frameworks and the stress caused by economic insecurity [67]. The confluence of social marginalisation, limited access to official services, and pervasive unemployment can cause a complex interplay of trauma, poverty, and depression among children [68].

Cognitive behavioural therapy has been shown to work in high-income environments and in children. Nevertheless, it is essential to note that in low-income contexts, significant disparities exist in allocating resources and accessibility to mental health services [69]. Consequently, implementing conventional mental healthcare models becomes impractical in such circumstances.

The outcomes of our study indicate that cognitive behavioural therapies must address both the root causes of exposure to violence and early trauma concurrently [70].

This study has several limitations. It should be noted that systematic reviews can result in fragmented evidence, which limits their ability to give adequate research information. Because this evaluation’s eligibility requirements are sensitive, a small but focused research group was chosen—furthermore, the included studies used samples that were largely male and Caucasian. As a result, this research may not represent different nationalities, cultures, or socioeconomic circumstances. These findings are limited in generalisability and applicability beyond the indicated situations. Many factors must be considered when adapting first-world outcomes to third-world settings.

## Figures and Tables

**Table 1:** Databases, used index terms, and Boolean search words.

Databases	Key Search Words
1. Ebscohost	Childhood And trauma And interventions AND exposure And violence AND trauma And interventions And children.
2. Science Direct	Childhood And trauma And interventions AND exposure And violence AND trauma And interventions And children.
4. PubMed	Childhood And trauma And interventions AND exposure And violence AND trauma And interventions And children.
5. Google Scholar	Childhood And trauma And interventions AND exposure And violence AND trauma And interventions And children.
6. PsycINFO	Childhood And trauma And interventions AND exposure And violence AND trauma And interventions And children.

**Table 2:** CASP for selected studies.

Author and Year	Title	Study Appraisal
Gilbert et al. [21]	The Use of Psychological First Aid in Children Exposed to Mass Trauma Results Recent research on Psychological First Aid (PFA) demonstrates its favourable reception among young individuals, families, and healthcare providers. Moreover, it has shown associations with reductions in depressive and post-traumatic stress symptoms, enhanced self-efficacy, increased awareness of disaster preparedness and recovery, and improved feelings of safety and connectedness. Notably, the modular PFA approach’s adaptability and cultural adjustments have emerged as noteworthy themes.	90%
Woollett et al. [22]	Trauma-informed art and play therapy: Pilot study outcomes for children and mothers in domestic violence shelters in the United States and South Africa Results Initially, the children exhibited elevated symptoms of potential depression and post-traumatic stress disorder (33% and 66%, respectively). After the study, there was a significant decrease in depressive symptoms (mean reduction from 13.7 to 8.3, *p* = 0.01). Additionally, a non-significant trend indicated potential improvement in PTSD symptoms (reduction from 40.0 to 34.4, *p* = 0.21). The children disclosed that the art medium facilitated their ability to articulate challenging emotions and experiences related to their mothers. Several children reported that it helped them in managing problematic behaviours.	80%
Ortiz et al. [23]	The Role of Mindfulness in Reducing the Adverse Effects of Childhood Stress and Trauma Results Well-designed and systematic mindfulness interventions positively impact young individuals’ mental, behavioural, and physical well-being. Additionally, when considering the outcomes seen in adults who have undergone extensively studied interventions, there is the potential for preventing the adverse health consequences linked to childhood trauma exposure. Future endeavours should focus on refining the implementation of these high-quality mindfulness programs among youth. Subsequent research should delve into the mechanisms underlying mindfulness and its long-term effects, from childhood through adulthood and potentially extending to future generations.	80%
Woods-Jaeger et al. [24]	The Art and Skill of Delivering Culturally Responsive TF-CBT in Tanzania and Kenya Results The results of this paper support the notion that Trauma-Focused Cognitive Behavioral Therapy is well-received and suitable as an approach to enhancing children’s mental health. Gaining a more comprehensive insight into the methods employed by non-specialist counsellors to ensure that the treatment aligns with the cultural and socioeconomic backgrounds of the participants can offer valuable insights for guiding the implementation of future evidence-based therapies.	80%
Meentken et al. [25]	Eye movement desensitization and reprocessing (EMDR) in children and adolescents with subthreshold PTSD after medically related trauma: design of a randomized controlled trial. Results Given the limited available data on the efficacy of EMDR in children who have experienced medically related trauma, clinicians, researchers, and young patients receiving care in hospitals can find value in this research study.	50%
Stewart et al. [26]	Implementing Evidence-Based Psychotherapy for Trauma-Exposed Children in a Lower-Middle Income Country: The Use of Trauma-Focused Cognitive Behavioral Therapy in El Salvador. Results The outcomes revealed substantial effect sizes in reducing trauma symptoms as reported by youth (Cohen’s d = 2.04), as well as in decreasing depressive symptoms (Cohen’s d = 1.68) and anxiety symptoms (Cohen’s d = 1.67). Our assessment of the program indicates that it was viable to instruct healthcare providers in Trauma-Focused Cognitive Behavioral Therapy (TF-CBT), and these providers, in turn, could deliver TF-CBT within community-based settings. Furthermore, TF-CBT proved to be an effective treatment option for addressing trauma-related issues in the youth population of El Salvador. This initiative represents a pivotal initial stride in disseminating and implementing evidence-based trauma-focused interventions for young individuals in Latin American nations.	60%
Tabone et al. [27]	Trauma-informed intervention with children: integrating the CANS Assessment with the ARC Framework in a Clinical Setting Results The results carry important implications for clinicians, emphasising the value of incorporating CANS-ARC mapping into the assessment and treatment procedures. Additionally, they underscore the significance of collaborative efforts across different systems to ensure consistent care for children who have encountered trauma. The research revealed a noteworthy decline in trauma-related symptoms across all ARC outcome categories over time, even after accounting for basic demographic variables. This study contributes to evidence regarding treating trauma-related symptoms in children, particularly with the practical application of the CANS-ARC mapping approach within a real-world clinical context. The implications of these findings are substantial, highlighting the need for clinicians to seamlessly integrate assessment and treatment using the CANS-ARC mapping method and fostering collaboration across systems to provide continuous care for children who have undergone traumatic experiences.	50%
Fazel et al. [28]	Five applications of narrative exposure therapy for children and adolescents presenting with post-traumatic stress disorders Results The cases are examined in terms of how the NET lifeline played a role in encouraging participation in treatment, making practical adjustments for individuals with intellectual disabilities, and adapting NET, which requires relatively brief training for healthcare professionals, to various situations and manifestations. The discussion emphasises the significance of enhancing access to care to ensure that young individuals receive support for their most challenging and disruptive memories.	60%
Dorsey et al. [29]	Effectiveness of task-shifted trauma-focused cognitive behavioural therapy for children who experienced trauma. Results A 640-child research included 320 girls and 320 boys. With a standard deviation of 1.6 years, the children averaged 10.6 years old. In three out of four sites, Trauma-Focused Cognitive Behavioural Therapy (CBT) treated post-traumatic stress (PTS) better than usual care (UC) in three-month randomised clinical research. In rural and urban Kenya, Trauma-Focused Cognitive Behavioral Therapy (TF-CBT) outperformed usual care (UC) after 12 months. At 12 months, Trauma-Focused Cognitive Behavioral Therapy (TF-CBT) and usual treatment did not significantly differ in improvement rates for children in Tanzania. In both rural and urban Tanzania, the findings were consistent. Secondary outcomes showed a similar tendency, with heavier implications in Kenya. Children suffer increased stress and difficulty due to food deprivation, lower guardian health, and greater exposure to traumatic events.	80%
Danielson et al. [30]	Reducing substance use risk and mental health problems among sexually assaulted adolescents: a pilot randomized controlled trial. Results The results showed that RRFT patients had higher levels of PTSD, depression, and general internalising symptoms than TAU patients. Nevertheless, it is crucial to use caution when interpreting results from between-group comparisons due to the substantial changes in baseline functioning observed between the two conditions. In contrast, the success of the RRFT adolescents is assessed based on the replication of feasibility outcomes and the observed enhancements within the group over some time.	70%
Deblinger et al. [31]	Trauma-focused cognitive behavioural therapy for children: impact of the trauma narrative and treatment length Results The mixed-model analysis of covariance (ANCOVA) results indicated significant improvements in 14 outcome variables across all conditions after therapy. Regarding individual outcomes, there were notable differences in the main and interaction effects seen among conditions.	80%
Katsonga-Phiri et al. [32]	Trauma Intervention in Sub-Saharan African Children: A Systematic Literature Review Results The studies that were assessed exhibited limitations in terms of methodology and intervention. Several studies have identified three prominent limitations. The limitations encompassed in this study consist of the utilisation of limited sample sizes, the absence of a control group, and the need for long-term follow-up.	90%
Lokuge et al. [33]	Mental health services for children exposed to armed conflict: Me’decins Sans Frontie‘res’ experience in the Democratic Republic of Congo, Iraq and the occupied Palestinian territory. Results A total of 3025 20-year-olds presented to MSF mental health services in DRC, Iraq, and oPt between 2009 and 2012, representing 14%, 17.5%, and 51% of all presentations. Sexual violence in DRC (36.5%), domestic violence in Iraq (17.8%), and incarceration or detention in oPt (33%) were the main causes. DRC, Iraq, and oPt youth reported 25.9%, 55.0%, and 76.4% armed conflict-related precipitants. Children and teenagers often presented with anxiety, followed by mood, behaviour, and somatisation issues, which varied by nation and precipitating event. Even though 45.7% left programs early, 97% of individuals who finished care indicated improvement in their complaints.	70%
Ford et al. [34]	Randomized trial comparison of emotion regulation and relational psychotherapies for PTSD with girls involved in delinquency. Results The sample size is relatively small. The dropout rate is relatively high. All measurements were based on self-report data, and the assessor was not blinded. There is no equivalent to a thoroughly validated treatment for post-traumatic stress disorder (PTSD). TARGET had higher initial post-traumatic stress disorder (PTSD) symptoms, namely criteria B symptoms, than ETAU. There was a loss of participants during the follow-up examination, which was limited explicitly to female individuals.	60%
Ray et al. [35]	Child-centred play therapy and adverse Childhood experiences: A Randomized Controlled Trial. Results According to the findings of a repeated measures linear mixed model, children who participated in the CCPT program exhibited statistically significant improvements in empathy, social competence, and self-regulation/responsibility. Additionally, these children showed a decrease in total behaviour problems. The findings of this study demonstrate the efficacy of Cognitive Coping and Processing Therapy (CCPT) in addressing the needs of children who have experienced adverse childhood experiences (ACE) and are at risk of developing complex trauma.	100%
Morison et al. [36]	Effectiveness of creative arts-based interventions for treating children and adolescents exposed to traumatic events: a systematic review of the quantitative evidence and meta-analysis. Results The findings of this study indicate that the implementation of arts-based therapies led to a substantial decrease in scores related to the symptoms of post-traumatic stress disorder (PTSD) compared to pre-intervention measures. This conclusion is supported by data from 15 studies, which yielded a standardised effect size (g) of −0.67, with a *p*-value of less than 0.001. Furthermore, compared to a control group, arts-based therapies showed a notable reduction in PTSD symptom scores across seven studies, resulting in a standardised effect size (g) of −0.50, with a *p*-value of less than 0.001. The manifestation of challenges and anxiety in an external manner yielded varied outcomes. However, there was a notable reduction in negative affect.	90%
Malhi et al. [37]	Using innovative narrative therapies with children who witness intimate partner violence. Results The significant interventions involved requesting children to sketch, compose poetry, and narrate stories about their family and personal circumstances. The individuals effectively expressed their powerlessness and indignation upon seeing these distressing occurrences. Over time, all three somatic symptoms were resolved by addressing their concerns inside a secure environment. The healing process changed several forms of artistic expression, including visual art, poetry, and written or spoken narratives.	80%
Pernebo et al. [38]	Reduced psychiatric symptoms at 6- and 12-month follow-up of psychotherapeutic and psychoeducative group interventions for children exposed to intimate partner violence. Results Significant and lasting improvements were observed in the treatment outcomes of children, specifically in their internalising and externalising symptoms, as well as symptoms related to traumatic stress. These improvements were evident from the post-treatment assessments and continued to be present during the follow-up assessments. The statistical analysis revealed a significant effect (*p* = 0.004–0.044), with effect sizes ranging from 0.29 to 0.67. There was no notable escalation in symptoms as reported. Furthermore, there was a noteworthy scarcity of documented instances where children were exposed to violence on an ongoing or recurring basis.	80%
Mannarino et al. [39]	Trauma-Focused Cognitive-Behavioral Therapy for Children: Sustained Impact of Treatment 6 and 12 Months Later. Results The follow-up assessments conducted at 6 and 12 months demonstrated the continued effectiveness of TF-CBT treatment, affirming the study’s hypothesis. This held true regardless of whether children and their parents underwent 8 or 16 treatment sessions or if they actively constructed a trauma narrative (TN) and discussed it with the therapist. These results align with prior research indicating that the therapeutic benefits of TF-CBT endure for 1 to 2 years.	90%

**Table 3:** Critical appraisal table.

Study Authors	Title	Sample	Objectives	Study Design	Study Setting	Limitations
Gilbert et al. [21]	The Use of Psychological First Aid in Children Exposed to Mass Trauma	7 to 16 years	The dissemination and promotion of Psychological First Aid (PFA) as an intervention to facilitate short-term coping and long-term functioning in the aftermath of disasters.	Systematic review	USA	There is a need for more empirical research that investigates the influence of PFA on the mental well-being of young individuals.
Woollett et al. [22]	Trauma-informed art and play therapy pilot study outcomes for children and mothers in domestic violence shelters in the United States and South Africa	6–14 years	To explore the effects of a pilot intervention combining trauma-focused cognitive behavioural therapy (verbal) with play therapy (non-verbal).	Pilot study	USA and SA	The study was not experimental, making it challenging to assess whether the intervention impacted mental health symptoms. The sample size was also small; qualitative findings should be interpreted cautiously.
Ortiz et al. [23]	The Role of Mindfulness in Reducing the Adverse Effects of Childhood Stress and Trauma.	11.5–15 years	Examining high-quality structured mindfulness instruction may mitigate the negative effects of stress and trauma related to adverse childhood exposure, improving short- and long-term outcomes and potentially reducing poor health outcomes in adults.	Systematic review	USA	Collectively, the findings of this study indicate that the provision of well-designed mindfulness training can alleviate the detrimental consequences of stress and trauma resulting from unpleasant childhood experiences. This can enhance immediate and prolonged results, conceivably diminishing unfavourable health outcomes during adulthood. Further research is required to improve the execution of mindfulness programs targeted towards young individuals and to investigate the long-term effects extending into adulthood.
Woods-Jaeger et al. [24]	The art and skill of delivering culturally responsive trauma-focused cognitive behavioural therapy in Tanzania and Kenya.	7–13 years	This study examines the facilitators, barriers, and methods employed in implementing evidence-based trauma interventions within the context of mental health services.	Qualitative study	Kenya and Tanzania	The sample size was small, and differences by country were not examined. TF-CBT’s effectiveness has yet to be discovered in Kenya and Tanzania due to its impact on language.
Steward et al. [26]	Implement evidence-based psychotherapy for trauma-exposed children in a low-middle-income country. The use of TF-CBT therapy	3–18 years	El-Salvador TF-CBT provider training. Program evaluation to establish implementation feasibility and treatment efficacy.	Qualitative study	Switzerland	The study did not utilise a controlled trial design, and its primary aim was not to conduct a stringent research investigation. Instead, its focus was on facilitating the training and adoption of TF-CBT among local healthcare providers.
Tabone et al. [27]	Trauma-informed intervention with children integrating the CANS assessment with the ARC framework in a Clinical setting	12 years	Examines using the attachment, self-regulation, and competency (ARC) model to guide intervention for child trauma has clinical benefits.	Longitudinal study	USA	The drawback of this study is its predominant focus on a single racial and ethnic group, which limits its diversity. Consequently, the findings may not broadly apply to other states or regions.
Meentken et al. [25]	Eye movement desensitisation and reprocessing (EMDR) in children and adolescents with sub-threshold PTSD after medically related trauma: design of a randomised controlled trial	12–15 years	This study aims to determine if standardised eye movement desensitisation and reprocessing (EMDR) therapy reduces post-traumatic stress symptoms (PTSS) in children with sub-threshold PTSD after hospitalisation.	Randomised trial	Netherlands	This study was conducted at a single medical centre, Erasmus MC, where EMDR sessions were exclusively administered. Nevertheless, participants were drawn from various regions across The Netherlands, which enhances the applicability of the study’s results to a broader context.
Fazel et al. [28]	Five applications of narrative exposure therapy for children and adolescents presenting with post-traumatic stress Disorders	14 years	This study explores NET integration into clinical practice, expanding its use in routine medical practice.	Case study		Five situations show NET’s potential to integrate clinical practice into everyday practice. The evidence base for NET needs improvement due to RCT shortcomings such as small sample sizes, non-active control groups, limited follow-up, and focus on refugees and asylum seekers.
Dorsey et al. [29]	Effectiveness of task-shifted trauma-focused cognitive behavioural therapy for children who experienced parental death and post-traumatic stress in Kenya and Tanzania	13 years	Trauma-focused cognitive-behavioural therapy (TF-CBT) is tested for its ability to reduce post-traumatic stress (PTS) symptoms in Kenyan and Tanzanian children who have lost a parent. This study will examine how TF-CBT affects other mental health symptoms and whether task shifting is feasible using experienced, local lay counsellors as trainers and supervisors.	A randomised clinical control trial	Kenya and Tanzania	The study employed a single-blind design in which interviews were coded according to the assigned condition. Nevertheless, participants were cognizant of their group assignment, potentially leading to disclosing information or developing biased expectations associated with their respective assignments.
Mannarino et al. [39]	Trauma-Focused Cognitive-Behavioral Therapy for Children: Sustained Impact of Treatment 6 and 12 Months Later	4–11 years	The study assessed the effectiveness of trauma-focused cognitive behaviour therapy (TF-CBT) as delivered by the community in comparison to standard community treatment for children exhibiting symptoms of post-traumatic stress disorder (PTSD) resulting from intimate partner violence (IPV).	A randomised control trial	USA	Following the central hypothesis of this research, the results obtained from our follow-up assessments conducted at 6 and 12 months unequivocally show that the benefits of TF-CBT treatment were enduring. This remained consistent regardless of whether children and parents underwent 8 or 16 treatment sessions or whether the children actively participated in creating a trauma narrative (TN) and discussing it with the therapist. Furthermore, these findings align with prior studies, which have consistently shown that improvements achieved through TF-CBT are maintained for 1 to 2 years after the conclusion of treatment.
Danielson et al. [30]	Reducing substance use risk and mental health problems among sexually assaulted adolescents: a pilot randomised controlled trial	13–17 years	This study aims to assess the viability and effectiveness of Risk Reduction via Family Therapy (RRFT) in mitigating the risks associated with substance use and trauma-induced mental health issues among adolescents who have experienced sexual assault.	Randomised trial	USA	There are fundamental disparities in various aspects between RRFT (Recovery-Oriented Residential Treatment) and TAU (Treatment as Usual) and variations in the treatment dosage provided. The sample size is limited in scope. The inclusion criteria employed in this study are characterised by their lack of restrictions—the presence of heterogeneity within the sample.
Ford et al. [34]	Randomised trial comparison of emotion regulation and relational psychotherapies for PTSD with girls involved in delinquency	13–17 years	This study compared the effects of Trauma Affect Regulation: Guide for Education and Therapy (TAR-GET) and Enhanced Treatment as Usual (ETAU) on 59 delinquent 13–17-year-old girls. These participants met full or partial PTSD criteria.	Randomised trial	USA	Small sample size. Dropouts were high. The assessor was not blinded and performed all assessments using self-report data. Nothing beats a well-validated PTSD treatment. TARGET showed higher initial criterion B PTSD symptoms than ETAU. Female participation dropped throughout the follow-up.
Katsonga-Phiri et al. [32]	Trauma Intervention in Sub-Saharan African Children: A Systematic Literature Review	0–19 years	This paper aims to serve as a foundational resource for critically assessing trauma therapies now applied to children in sub-Saharan Africa. This encompasses children residing in conflict-ridden and conflict-free nations, as children are susceptible to experiencing trauma due to parental loss due to chronic disease, poverty, and various manifestations of violence.	Systematic review	Southern Africa	The examined studies had methodological and interventional limitations. Three common problems in research were small sample sizes, the lack of a control group, and the lack of long-term follow-up. However, other publications met most criteria except for defining youth as ending at age 25. The studies were included despite their difficulty in determining which outcomes applied to youth and not to 20–25-year-olds
Lokuge et al. [33]	Mental health services for children exposed to armed conflict: Me’decins Sans Frontie‘res’experience in the Democratic Republic of Congo, Iraq and the occupied Palestinian territory	15–19 years	This analysis sought to (i) describe the demographics of children and adolescents presenting to these programs, (ii) describe their mental health complaints and precipitating or underlying events, (iii) compare the above factors across the three countries in this analysis, and (iv) describe the mental health services provided and their short-term outcomes in each country.	Comparative study	DRC, Iraq, and Palestine	Programs that did not specifically focus on children and adolescents had relatively few young individuals seeking mental health services. Enhancing the participation of children and adolescents can be achieved by disseminating mental health service information tailored to their needs, engaging in community-based outreach efforts, and establishing connections with other sectors within the healthcare system to address specific exposures.
Deblinger et al. [31]	Trauma-focused cognitive behavioural therapy for children: impact of the trauma narrative and treatment length	4–11 years	This study examined the effects of Trauma-Focused Cognitive Behavioral Therapy (TF-CBT) with and without the trauma narrative (TN) component over 8 and 16 sessions.	Randomised control trial	Pennsylvania	The condition consisting of eight sessions, incorporating the TN component, appeared to be the most optimal and proficient approach for mitigating parental distress specific to abuse and reducing children’s dread connected to abuse and overall anxiety. In contrast, parents assigned to the 16-session, no-narrative condition exhibited significant improvements in effective parenting practices and reduced externalising child behavioural problems after completing the treatment.
Ray et al. [35]	Child-centred play therapy and adverse childhood experiences: A randomised controlled trial		This randomised controlled trial examined how child-centred play therapy improved social and emotional skills and reduced behavioural disorders in children with two or more adverse childhood experiences.	Randomised control trial		Based on the analysis using a repeated measures linear mixed model, it was observed that children undergoing CCPT exhibited statistically significant improvements in empathy, social competence, and self-regulation/responsibility, alongside notable reductions in overall behaviour problems. These findings indicate the effectiveness of CCPT in benefiting children who have experienced adverse childhood experiences (ACE) and are at risk of complex trauma.
Morison et al. [36]	Effectiveness of creative arts-based interventions for treating children and adolescents exposed to traumatic events: a systematic review of the quantitative evidence and meta-analysis.	8–16years	This study seeks to analyse the available data about therapies utilising creative arts to alleviate psychological distress in individuals who have experienced traumatic events.	A systematic review of the quantitative evidence and meta-analysis		Arts-based therapies led to a notable reduction in PTSD symptom scores when compared to baseline (as evidenced by 15 studies, g = −0.67, *p* < 0.001) and when compared to a control group (as indicated by seven studies, g = −0.50, 0 < 0.001). While the outcomes for externalising difficulties and anxiety varied, there was a significant decrease in negative mood.
Malhi et al. [37]	Using innovative narrative therapies with children who witness intimate partner violence	11–12 years	This case series aims to show how trauma creative narrative interventions like poetry and storytelling help children communicate their anxieties and suffering in a safe space.	Case study	India	Children were asked to sketch, write poetry, and narrate tales about their families and situations as the main interventions. They were able to convey their powerlessness and outrage from seeing these horrible incidents. Over time, facing their concerns in a safe atmosphere resolved all three somatic symptoms. Art, poems, and written/oral narrations changed with this healing.
Pernebo et al. [38]	Reduced psychiatric symptoms at 6- and 12-months’ follow-up of psychotherapeutic and psycho-educative group interventions for children exposed to intimate partner violence	4–13 years	The present study investigated the long-term benefits of two proven group therapies specifically developed for children who have experienced intimate partner violence and their non-offending parent.	Longitudinal study	Sweden	Consistent and lasting improvements in children’s internalising and externalising symptoms and in traumatic stress symptoms were observed from the end of treatment through the follow-up assessments (*p* = 0.004–0.044; d = 0.29–0.67). There was no significant indication of symptom escalation. Furthermore, there were minimal reports of ongoing or renewed exposure to violence among the children.

**Table 4:** Themes emerging from selected studies.

Themes
1. Effectiveness and implementation of trauma intervention
2. Trauma intervention implementation
3. Barriers and facilitators of trauma
4. The psychological burden of trauma in children
5. Coping strategies of children exposed to trauma

**Table 5:** Results.

Author	Year	Intervention	Outcome
Woods-Jaeger et al. [24]	2017	TF-CBT	Positive outcome
Ray et al. [35]	2022	Child-centred play therapy	Positive outcome
Morison et al. [36]	2022	Art-based therapy	Positive outcome
Malhi et al. [37] and Fazel et al. [28]	2020, 2022	Narrative therapy	Positive outcome
Pernebo et al. [38]	2019	Psychoeducation	Positive outcome
Mannarino et al. [39]	2014	TF-CBT	Positive outcome

## Data Availability

Data sharing does not apply to this article, as no new data were created. The data used for this study are readily available; consult the reference list.
